# Functional morphology of the copulatory organs of a reed beetle and a shining leaf beetle (Coleoptera: Chrysomelidae: Donaciinae, Criocerinae) using X-ray micro-computed tomography ^*^

**DOI:** 10.3897/zookeys.547.7143

**Published:** 2015-12-17

**Authors:** Michael Schmitt, Gabriele Uhl

**Affiliations:** 1 Ernst-Moritz-Arndt-Universität, Allgemeine & Systematische Zoologie, Anklamer Str. 20, 17489 Greifswald, Germany

**Keywords:** Aedeagus, endophallus, flagellum, bursa copulatrix, spermatheca, sperm transfer

## Abstract

For more than 100 years it has been known that the sclerotised median lobe of beetles harbours a membranous structure (the "internal sac" or "endophallus") which is everted during copula inside the female genital tract. In order to explore the functional role of this structure and those associated with it, we cryofixed copulating pairs of *Donacia semicuprea* and *Lilioceris lilii* and studied the relative position of the elements of the copulatory apparatus of males and females by micro-computer-tomography.

We found that the everted endophallus fills the lumen of the bursa copulatrix completely. Our data suggest that in *Lilioceris lilii* the tip of the sclerotised distal part of the ejaculatory duct, the flagellum, is positioned exactly over the opening of the spermathecal duct inside the bursa copulatrix. The mouth of the bursa copulatrix in *Donacia semicuprea* is armed with a strong muscle ring, and the whole wall of the bursa is covered externally with a layer of muscle fibres. These morphological differences correspond with differences in mating behaviour: In reed beetles (Donaciinae), females seemingly can control mating to a higher degree than in lily beetles (*Lilioceris* spp.).

## Introduction

As the primary role of copulatory organs is to secure transfer of sperm from males to females, they could, in principal, be shaped very simply. A tube, rigid or elastic, and a corresponding basket would do. The fact that copulatory organs are often complex and species-specific has traditionally been explained as a lock-and-key device that guarantees the preservation of the species and prevents waste of time, energy, and sperm by copulations between allospecific partners (Shapiro and Porter 1989). Only since Eberhard’s seminal book on "Sexual Selection and Animal Genitalia" (1985) zoologists have learned to interpret the morphology of genitalia in terms of individual fitness maximisation. This paradigm explains why shape and function of male and female copulatory organs are normally species-specific, and it provides a framework for understanding the functional role of peculiar structural elements of the copulatory apparatus.

**Figure 1.**
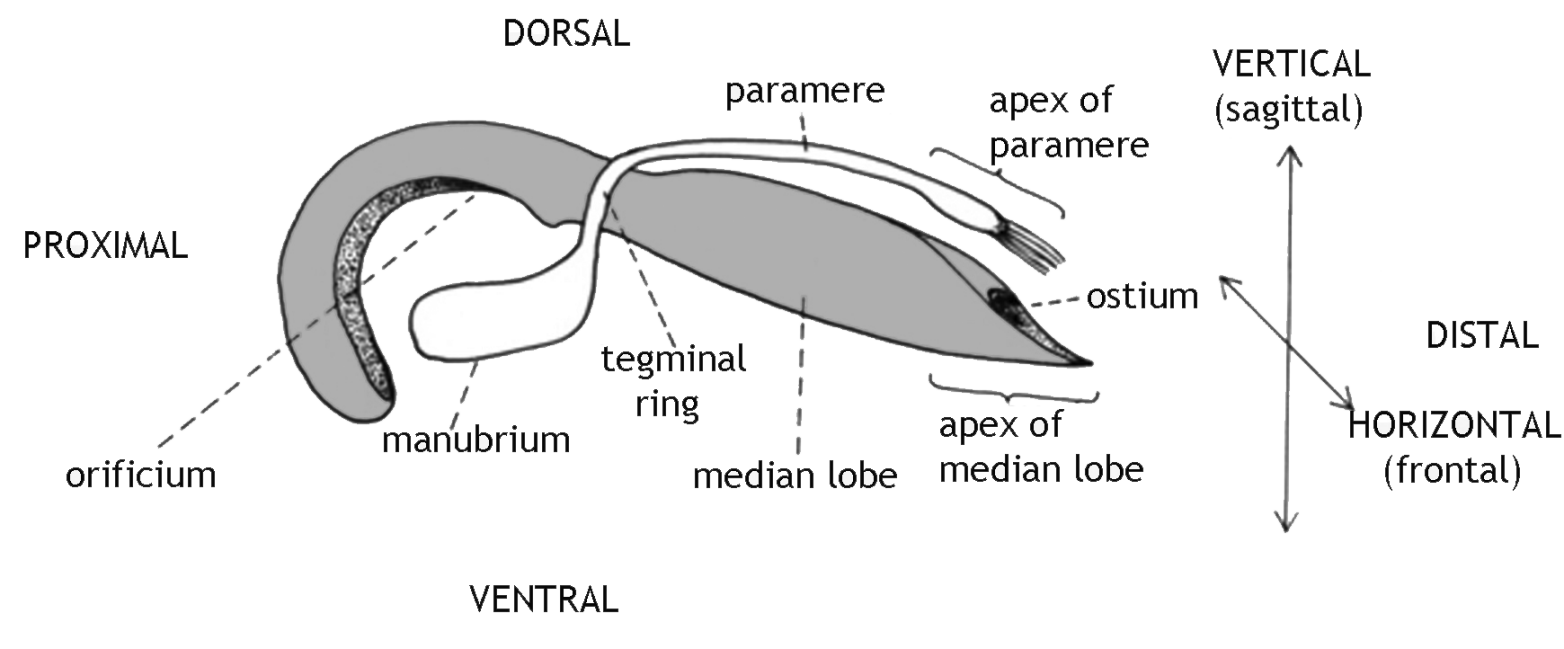
Schematic drawing of the sclerotised part of a Donaciinae aedeagus. The given terms of orientation do not refer to the position within the male abdomen nor within the female during copulation since these structures are rotated during mating. The median lobe is shaded grey. Paramere, tegminal ring and manubrium compose the tegmen.

In beetles, the form of the male copulatory apparatus (aedeagus), especially its median lobe, has found the lively interest of taxonomists (e.g., Kraatz 1881; Weise 1889a and b; Sharp and Muir 1912). Crowson (1955: 114) introduced the basic terminology and a hypothesis on the evolutionary transformation of what he called the "complete cucujoid" aedeagus. According to Crowson, this organ consists of a median lobe – functionally a sclerotised tube, mostly bent, often termed "penis" – and attached elements (see Fig. 1). These latter elements are basically a sclerotised ring around the median lobe from which proximal apodemes and distal parameres arise. We prefer "median lobe" because "penis" is, in our opinion, not a morphological but a functional term. It meant originally "the intromittant organ" (Tuxen 1970: 305). In many Cerambycidae the median lobe hardly, and if, then extremely briefly, enters the female body. On the other hand in, e.g., the sagrine *Mecynodera coxalgica* also the paramere is intromitted. Therefore, the term "penis" seems not just adequate throughout. In addition, we want to avoid any idea of homology of the median lobe and penes in other taxa. Consequently, we use the terminology of Kingsolver (1970) and Clark (1977), with the modification that we use "endophallus" for "internal sac" (following Burke 1959). Among Chrysomelidae, two types of aedeagi are present, those with parameres and those without. The morphology of the female copulatory organs has been largely neglected by taxonomists (an exception is, e.g., Döberl 1986).

However, only few investigators have studied the functional roles of the different elements of the male copulatory apparatus (e.g. Heberdey 1931, Cerezke 1964, LeCato and Pienkowski 1973, Rodriguez et al. 2004) and their interaction with the female copulatory organs during mating. Blunck (1912) studied the reproduction of *Dytiscus marginalis*, including detailed description of the relative position of the copulatory organs during mating and the formation of the spermatophore. Another meticulous investigation was done by Krautwig (1930), on the weevil *Sitophilus granarius*. Goldson and Emberson (1981) reported on the copulation of another weevil, *Hyperodes bonariensis*, and also described the relative position of male and female organs during sperm transfer. Flowers and Eberhard (2006) dissected mated pairs of 12 Neotropical leaf beetle species and described the coupling devices. For the present study, the most relevant publication is that by Harnisch (1915) who observed and dissected copulating pairs of several leaf beetle species and depicted the elements of the male and the female copulatory apparatus, isolated and in copula. He speculated that median lobe and the fused parameres act as a clasper organ during copulation in reed beetles (Chrysomelidae: Donaciinae). Düngelhoef and Schmitt (2006) showed that this idea is not congruent with observation of life beetles. The apex of the fused parameres hardly ever gets into physical contact with the female abdomen. Their tip is covered with mechanoreceptors and other sensilla, so that the functional role of the parameres is rather that of "genital feelers" (Düngelhoef and Schmitt 2010) than that of a coupling device.

During copulation, a membranous sac, the endophallus, is everted through the ostium of the median lobe (fig. 1) inside the female bursa copulatrix. The ejaculatory duct transverses the endophallus, ending in a sclerotized tube, the flagellum. In addition, the wall of the endophallus bears sclerites in several beetle species studied so far (Berti and Rapilly 1976, Askevold 1991, Hayashi 2005, Flowers and Eberhard 2006, Düngelhoef and Schmitt 2006).

In earlier papers, Düngelhoef and Schmitt (2006, 2010) argued that the mechanical footing between the mating partners during copulation is achieved by the male’s endophallus inflated inside the female’s bursa copulatrix. Shape and surface morphology of the external face of endophallus and bursa correspond, so that hemolymph pressure inside the endophallus and high friction between the two surfaces warrant a strong coupling. Exorbitant armour of the endophallus obviously serves additional roles, e.g. it imposes indirect costs to subsequent copulations of the female by injuring the bursa wall (see Crudgington and Siva-Jothy 2000 and the nearly 400 papers citing this publication, according to Google Scholar, last time checked September 10, 2015).

The mating behaviour of leaf beetles in general was described by Jolivet (1999), Bienkowski (1999) investigated on it in 14 palaearctic species of reed beetles. Konstantinov (2004) studied courtship, copulation and intrasexual competition of *Donacia crassipes* Fabricius, 1775. Here, males perform complex courtship behaviour and females can prevent or/and terminate copulation by kicking with their hind legs. In contrast, lily beetles do not show courtship behaviour. Males simply follow a female, mount when they reach her, and copulate. Females never show special defence behaviour, they either allow the male to mount or they escape. In some cases, females tried to get rid of a mounted male by kicking with their hind legs (Düngelhoef and Schmitt 2006).

We investigated cryofixed pairs of copulating leaf beetles. Male shining leaf beetles (Criocerinae) lack parameres while reed beetles (Donaciinae) possess a so-called complete cucujoid aedeagus. We focused especially on the relative position of male and female genitalia during copulation. We had observed earlier that copulating pairs of reed beetles quickly separate when disturbed, while copulating lily beetles (*Lilioceris lilii*) can only be separated by applying considerable force (Düngelhoef and Schmitt 2010). Thus, we expected to find morphological correlates of this behavioural difference.

## Material and methods

We collected numerous individuals of *Donacia semicuprea* Panzer, 1796 and *Lilioceris lilii* (Scopoli, 1763) in the area of Greifswald (northeast Germany). Copulating pairs were fixed using 70% ethanol at -12 °C (*Donacia semicuprea*) or liquid nitrogen (*Lilioceris lilii*). The fixed pairs were stored in 80% ethanol at -40 °C for at least ten days. We prepared them for X-ray micro-computed tomography (micro-CT) analysis by critical point drying (BAL-TEC CPD 030), glued them head downwards on the tip of a little plastic rod of 2 mm in diameter, with the tip of the female abdomen as close to the rotation axis as possible. Three pairs of *Donacia semicuprea* and two pairs of *Lilioceris lilii* were scanned under an Xradia Micro XCT-200 (Carl Zeiss X-ray Microscopy Inc.), using the 4× or 10× object lens units, at 30kV and 4W, with a pixel size of 5.36 µm or 2.34 µm. Tomography projections were reconstructed using the reconstruction software provided by XRadia. Volume rendering of image stacks was performed by using Amira 5.4.5 and Amira 5.6.0 (FEI Visualization Science Group, Burlington, USA) using the "Volren" or "Voltex" function.

We use the morphological terms as given in Fig. 1. Thus, "dorsal" does not refer to the back of a beetle but indicates the face of the aedeagus opposite the tegminal manubrium, while "distal" means the end of the median lobe bearing the ostium, i.e. the opening through which the endophallus is everted during copula and the sperm is finally leaving the male body. We use "aedeagus" addressing the complete male copulatory organ, i.e. the median lobe (= penis) plus the tegmen. The tegmen is either – in Criocerinae – v-shaped and attached to the ventral face of the median lobe, or – in Donaciinae – ring-shaped and surrounding the median lobe. Its basal part is formed as a vertical plate, the manubrium. In Donaciinae, the tegminal ring bears dorsally an unpaired projection pointing distad, the paramere.

## Results

**Figure 2.**
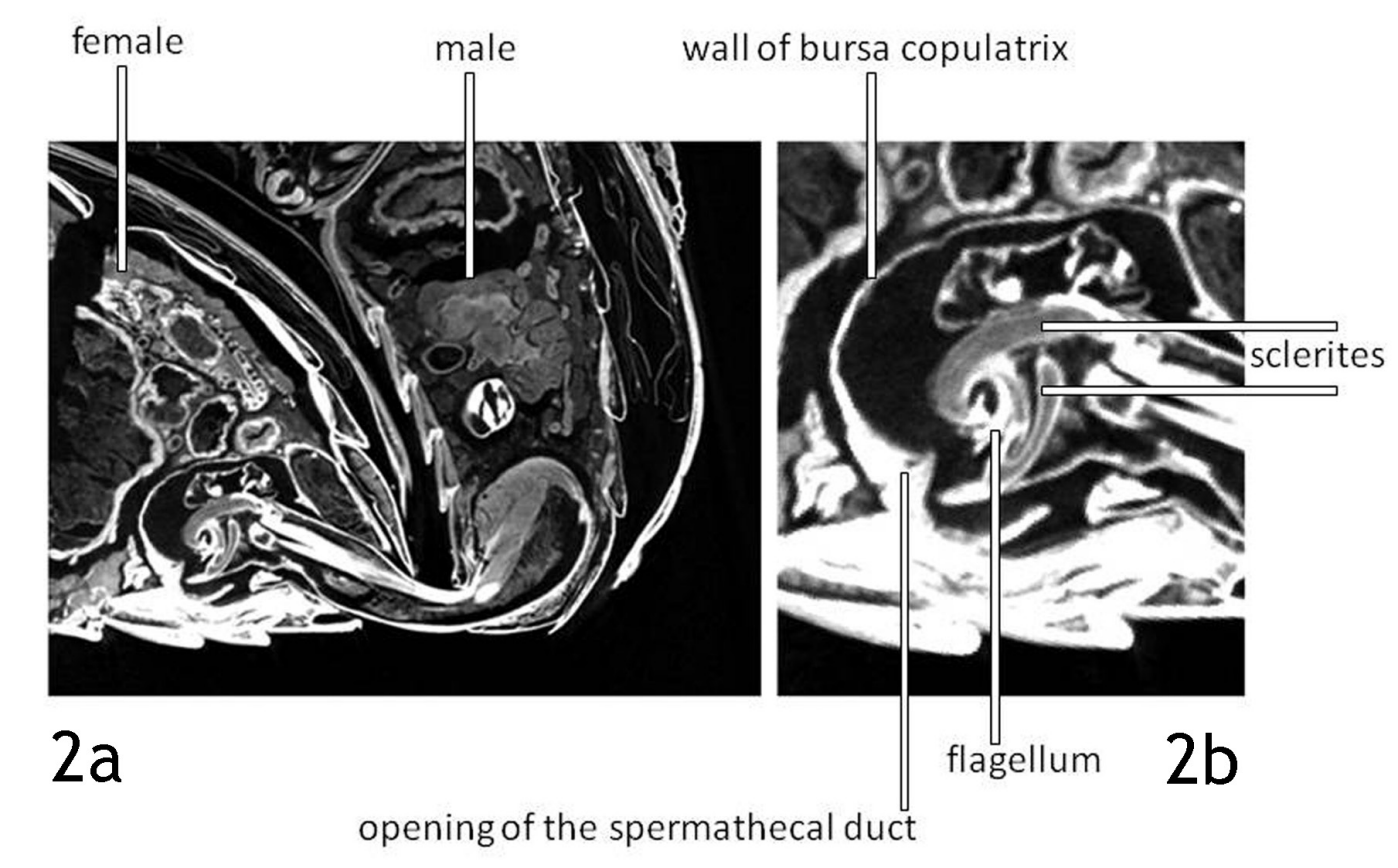
*Lilioceris lilii*. Virtual section – sagittal, median - through the abdomina of a mating pair. The endophallus is fully inflated (2a), the flagellum is positioned over the opening of the spermathecal duct (2b).

*Lilioceris lilii*: The median lobe of the aedeagus is inserted in the female abdomen, the endophallus is everted and inflated. We did not make an attempt to trace the ejaculatory duct because we focused on the relative position of male and female copulatory organs. Of the ejaculatory duct only the sclerotised distal part, the flagellum, gets into contact with the female body. The bursa copulatrix is nearly globular (length/height: 1.25/1), its opening is situated at half the length of the last sternite (Figs 2a, 4).

The tip of the small flagellum is positioned exactly opposite the opening of the spermathecal duct, see Fig. 2b. The spermathecal duct enters the bursa copulatrix through a sclerite that is embedded in the bursa wall. The gap between flagellum tip and duct opening in Figs 2a and 2b is most probably an artefact caused by shrinking of the tissue during fixation. The space between the wall of the inflated endophallus and the wall of the bursa copulatrix is probably also an artefact.

**Figure 3.**
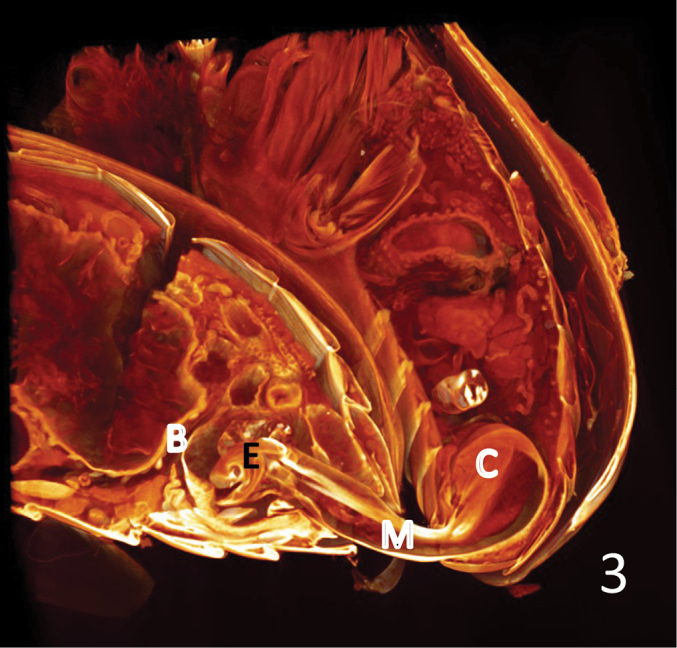
*Lilioceris lilii*, copulating pair. Volume rendering of the virtual sections right to the median, digitally stained. The terminal part of the spermathecal duct can be seen immediately left to the bursa wall. The shape of the bursa is nearly globular. B: bursa copulatrix; C: the compound muscle inserting at the manubrium and extending to the lateral rims of the basal orifice of the median lobe; E: endophallus; M: median lobe.

**Figure 4.**
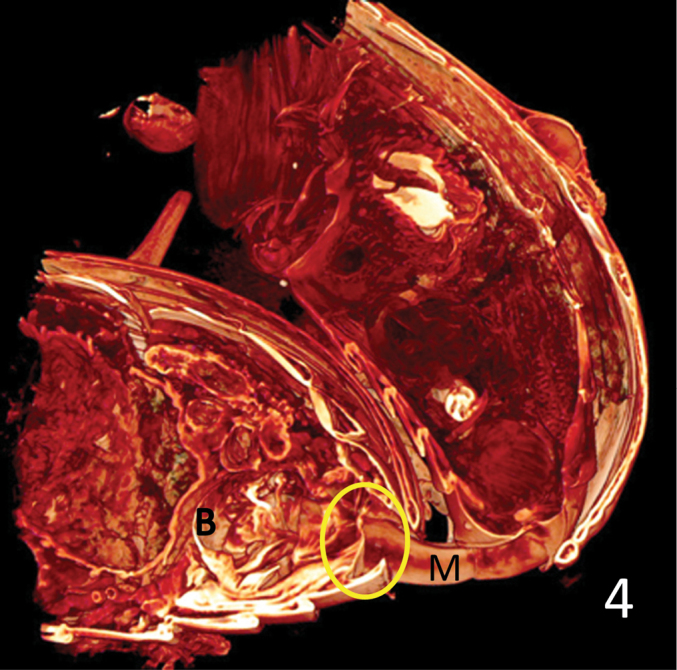
*Lilioceris lilii*, as Fig. 3, the opening of the bursa copulatrix and the adjacent part of the bursa wall (circle) do not show significant muscle layers but are mere membranes. B: bursa copulatrix; M: median lobe.

Fig. 3 shows the massive muscle under the orificium that makes the inflation of the endophallus when it contracts. On the ventral side of the median lobe we see the retractor tendon or muscle of the endophallus stretched through the whole length of the tube from the orificium to the ostium. The endophallus is fully everted through the ostium and fills the bursa copulatrix nearly completely. The opening of the bursa is membraneous and is strengthened only by few and delicate muscle fibres (Fig. 4, circle).

**Figure 5.**
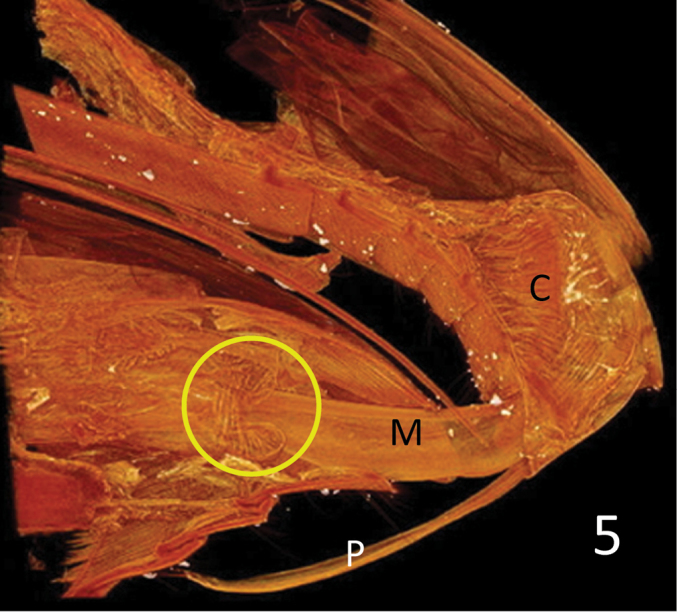
*Donacia semicuprea*. Volume rendering of the virtual sections – sagittal, right to the median – through the abdomina of a mating pair. The opening of the bursa copulatrix (circle) is armed with a conspicuous ring muscle. C: the compound muscle inserting at the manubrium and extending to the lateral rims of the basal orifice of the median lobe; M: median lobe; P: paramere.

*Donacia semicuprea*: The median lobe is inserted into the female body while the paramere remains outside (Fig. 5). The paramere is slender, its tip is bent towards the female abdomen. The paramere is in contact with the female abdomen only through its distal setae. We could not trace the shape and the measurements of the bursa copulatrix because in one pair the endophallus was not yet everted, and in the other two pairs the ovaries were so plump and massive that we could not discriminate the lining of the bursa and the tissue of the ovaries in the proximal part of the bursa. However, we could recognise that the bursa is considerably longer than in *Lilioceris lilii*.

**Figure 6.**
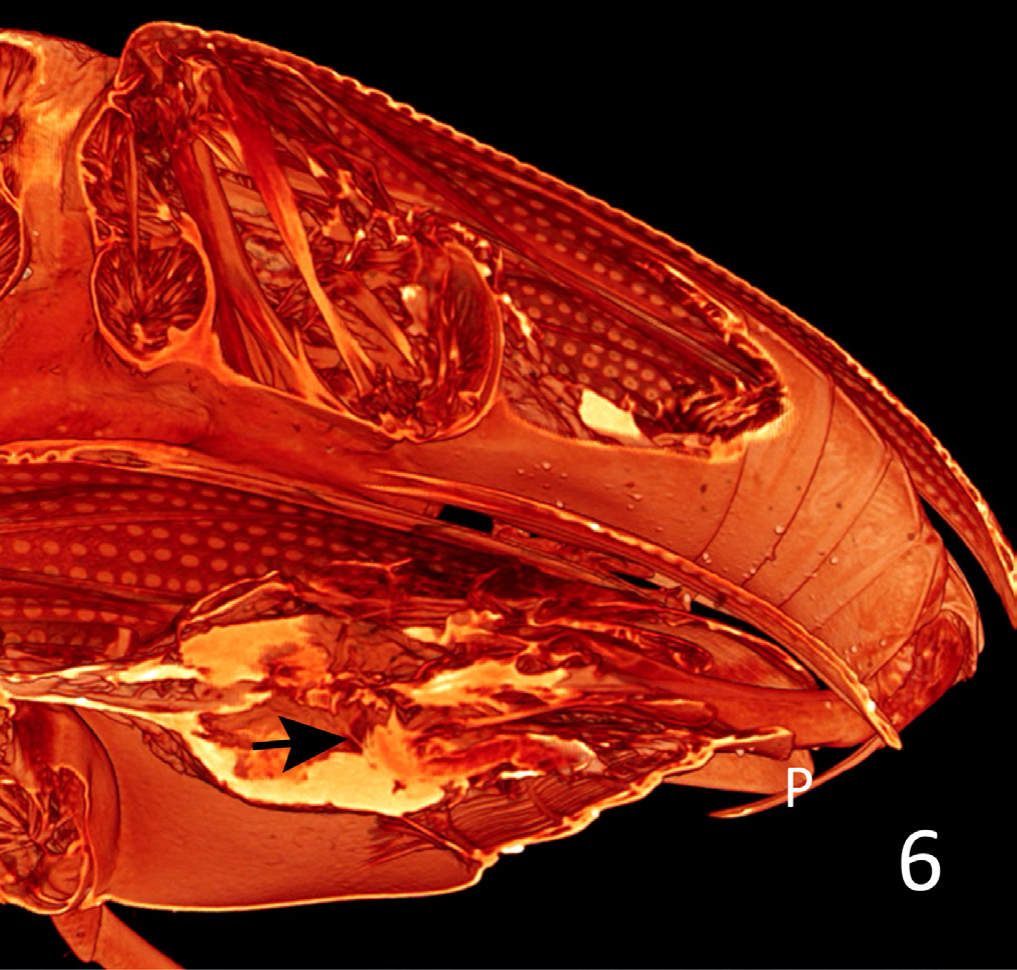
*Donacia semicuprea*, Volume rendering of ca. 390 virtual sections – sagittal, paramedian, tilted to the right – through the abdomina of a mating pair. The bursa is elongate, as seen from the proximal wall (arrow). The yellow shining areas inside the female abdomen are parts of the left ovary. P: paramere.

The tip of the median lobe is inserted into the bursa copulatrix. There is a strong muscular ring around the mouth of the bursa (Fig. 5, circle). Also, the outer surface of the wall of the bursa is covered with a layer of muscular fibres. The inflated endophallus seems to be longish, as is the bursa (Fig. 6). For the same reason as for the bursa we could not reconstruct the distal part of the endophallus.

## Discussion

When interpreting the morphological data, we have to take into account that the copulating partners may be fixed in different stages of, e.g. the intromission of the median lobe or endophallus inflation. Moreover, we cannot be certain that the interacting male and female copulatory organs remained exactly as they were in the millisecond when the beetles were cryofixed.

Already Krautwig (1930) found in paraffin sections of copulating pairs of *Sitophilus granarius* that the distal orifice of the ejaculatory duct was placed directly opposite the opening of the spermathecal duct inside the bursa copulatrix. Also Burke (1959 presumes that certain "sclerotized structures serve to effect a close connection between the gonopore of the male and the opening of the spermathecal duct of the female" in weevils. Consequently, we can only speculate that the spout-like opening of the flagellum (see fig. 12 in Düngelhoef and Schmitt 2006) is indeed put over the mouth of the spermathecal duct inside the bursa in *Lilioceris lilii*. The spermathecal duct opens into the bursa copulatrix through a sclerotised and thickened segment of the bursa wall (see fig. 49 in Berti and Rapilly 1976). We do not know whether or not the endophallus is constantly inflated inside the bursa during copulation. It might well be that the male everts and retracts the endophallus several times after intromission of the median lobe until the mating partners separate.

Our observation that the paramere of *Donacia semicuprea* remains outside the female body is in concordance with the earlier report of Düngelhoef and Schmitt (2010) and confirms the idea that it functions as a sense organ. Although there are species in which the males insert the parameres in the female body, e.g. the sagrine *Mecynodera coxalgica* (Düngelhoef and Schmitt 2010), it is highly unlikely that the males obtain mechanical footing that way. In nearly all species of Phytophaga studied to date, the parameres – if present at all – remain outside the female abdomen or remain inside the male body and are not even visible during copulation, as, e.g., in weevils and in longicorn beetles (Hubweber and Schmitt 2006, 2010, Düngelhoef and Schmitt 2006, 2010). Our observation that the inflated endophallus in *Lilioceris lilii* has the same shape as the inner space of the bursa, and that its surface bears denticles and spines (Düngelhoef and Schmitt 2006), together with the finding that the opening of the bursa and its walls do only bear few and delicate muscle fibres, correspond with an earlier observation (Düngelhoef and Schmitt 2010) that the copulating partners can be separated only by applying considerable force. Similar observations have been reported by Krautwig (1930) for *Sitophilus granarius*. In contrast, the fact that in *Donacia semicuprea* the inflated endophallus is longish and that the bursa bears a strong muscular ring around its opening and a marked layer of muscle fibres on its outer surface suggests that the female could be able to actively expel the male intromitting organ. This could explain the observation that it is difficult to cryofix copulating pairs in Donaciinae. Furthermore, Bienkowski (1999) and Jolivet (1999) report that females in Donaciinae play a pronouncedly active part in admitting males for mating as well as in terminating the copulation. It may well be that female donaciines can not only press out the male organ from the bursa but also prevent the male from intromitting his median lobe into the bursa by contraction of the sphincter muscle.

In species with a "complete cucujoid aedeagus", the compound muscle (Fig. 3: C, Fig. 5: C) that inserts at the manubrium and extends to the lateral rims of the basal orifice of the median lobe (the so-called ring-muscle, see Harnisch 1915), serves – at least – two different functions. One is to move the paramere, another is to extrude the endophallus during copulation by hemolymph pressure inside the "non-eversible part" of the endophallus (Kumar and Verma 1980). Thus, producing the necessary hemolymph pressure for the inflation of the endophallus has to be coordinated and possibly compromised with the independent movability of the parameres. Consequently, decoupling of these two functions requires differentiation of the "ring" muscle complex and independent neuronal control. Therefore, we speculate that the selective advantage of the loss of parameres in certain lineages of phytophagan evolution (Düngelhoef and Schmitt 2010) was that the massive "ring" muscle could be used exclusively to produce a high hemolymph pressure for the inflation of the endophallus. This compound muscle corresponds to the muscle labelled "RSP1... (retractor of the... tegminal apodeme)" by Kumar and Verma (1908). Kingsolver (1970) described the respective muscle in seed beetles (Chrysomelidae: Bruchinae) as "ventral muscle of the median lobe". In our opinion, it acts rather as an adductor than as a retractor.

The morphological difference between the two species corresponds with differences in mating behaviour. It suggests that in *Donacia semicuprea* – and probably in all donaciine species – females control admittance of males for mating and the duration of the copulation to a higher degree than in *Lilioceris lilii* (and probably in all Criocerinae).
